# Personality dimensions, depression, and eating behavior in individuals seeking bariatric surgery: a cluster analysis

**DOI:** 10.3389/fnut.2024.1429906

**Published:** 2024-08-13

**Authors:** Alessandro Rodolico, Valentina Lucia La Rosa, Caterina Romaniello, Carmen Concerto, Valeria Meo, Giulia Saitta, Serena Sturiale, Maria Salvina Signorelli, Ray Wang, Ramon Solhkhah, Catherine Phalen, Michael Kelson, Aguglia Eugenio, Stanley R. Terlecky, Florian Patrick Thomas, Fortunato Battaglia

**Affiliations:** ^1^Psychiatry Unit, Department of Clinical and Experimental Medicine, University of Catania, Catania, Italy; ^2^Unit of Psychodiagnostics and Clinical Psychology, University of Catania, Catania, Italy; ^3^PerFormat – Scuola di Specializzazione in Psicoterapia, Pisa, Italy; ^4^Williams College, Williamstown, MA, United States; ^5^Endeavor Health, Department of Psychiatry and Behavioral Sciences, Evanston, IL, United States; ^6^University of Chicago Pritzker School of Medicine, Chicago, IL, United States; ^7^Department of Medical Sciences, Hackensack Meridian School of Medicine, Nutley, NJ, United States; ^8^Department of Neurology, Hackensack Meridian School of Medicine, Nutley, NJ, United States

**Keywords:** bariatric surgery, personality traits, eating disorders, cluster analysis, depression

## Abstract

**Introduction:**

Psychiatric comorbidity is frequent in bariatric surgery candidates. This study aimed to classify bariatric surgery patients according to patterns of preoperative measures of the severity of the eating disorder (ED), depression, and personality traits.

**Methods:**

In the present cross-sectional study, 115 adult candidates for bariatric surgery (75 females, 65.22% of sample; mean age 37) were considered for analysis. Patients’ sociodemographic and psychopathological variables were collected. K-Means clustering analysis was adopted to classify bariatric surgery candidates according to their preoperative Eating Disorder Inventory-2 (EDI-2) scores. In addition, we assessed depression and personality traits using the Beck Depression Inventory-2 (BDI-2) and the Minnesota Multiphasic Personality Inventory-2 (MMPI-2).

**Results:**

Cluster analysis based on EDI-2 revealed two preoperative patterns: higher severity (*n* = 39), and low severity (*n* = 76). The more severe EDI-2 group had higher scores on the BDI-2 and presented higher scores on several MMPI-2 dimensions, particularly those related to anxiety (Psychasthenia, Anxiety, Fears, Obsessiveness), depression (Depression, including both content and clinical MMPI-2 subscales), externalizing symptoms (Anger, Cynicism, Type A Behavior), and social functioning (Social Introversion, Family Problems, Work Interference).

**Discussion:**

Eating disorders symptoms in candidates for bariatric surgery are closely related to depression and different psychological conditions assessed with MMPI-2. These psychological variables should be evaluated preoperatively and targeted with more specific psychological interventions.

## 1 Introduction

Obesity is a chronic condition characterized by an excessive accumulation of body fat (1), affecting almost one-third of adults worldwide, with minor geographical and demographic differences (2). The development of this condition is associated with biological, psychosocial, and behavioral factors (3, 4). Treatments are complex and often challenging (5). Previous studies suggest that bariatric surgery is an effective treatment that significantly reduces weight (6). However, it is invasive and carries some risks, and most patients regain weight after surgery and initial loss. Thus, to optimize care and predict which bariatric candidates will successfully achieve and maintain a healthy weight, it is important to investigate psychological factors associated with eating disorders in candidates for bariatric surgery. A wealth of research on preoperative psychological assessments has documented that depression and anxiety are linked to eating disorders in people with obesity (7, 8) and negatively influence patients’ quality of life (9). Of particular relevance is the interplay between depression, assessed with Beck Depression Inventory-2 (BDI-2) scale, eating disorders (10, 11), and outcome after bariatric surgery (12). Whether personality disorders are associated with eating disorders remains controversial (13). Both personality traits (avoidant and antisocial) and personality disorders (paranoid and schizotypal) were reported to be associated with eating disorders in medically treated obese patients (13). Moreover, in bariatric surgery candidates, high neuroticism, low extraversion, and low conscientiousness were associated with poor self-control in emotional eating (14) and with an increased probability long term weight regain after surgery (13). For instance, across studies, surgical candidates showed high impulsivity, which predicts a poor long-term outcome (15–17). The Minnesota Multiphasic Personality Inventory-2 (MMPI-2), a psychological test that assesses personality traits and psychopathology, has been extensively used (in its different versions) for preoperative evaluation of candidates for bariatric surgery. Patients were found to have compromised emotional control and a moderate increase in somatization, depression, and hypochondria scores (18). Their specific psychological scores predict outcomes (19, 20). Because literature indicates the incremental validity of the MMPI-2, relative to BDI-2 in the detection of depression and the reduction of misclassification errors when using both instruments (21), combining assessment instruments might improve preoperative evaluation in obese patients. Despite the extensive existing literature on personality characteristics in patients seeking bariatric surgery, little is known about subset of patients with specific personality traits, psychopathology, depression, and how these comorbidities relate to eating disorder symptoms. Thus, it is essential to investigate whether different patient sub-types exist in this population using an experimental approach to prevent over-reporting and under-reporting of depressive symptoms.

In the present study, we aimed to identify the most and the least severe psychological patient profiles among bariatric surgery candidates using cluster analysis, determining how these typical clusters relate to psychopathology, as measured by MMPI-2, and depression, as measured by the BDI-2.

## 2 Materials and methods

### 2.1 Study design and participants

The participants were candidates for various bariatric surgery procedures (Sleeve Gastrectomy, Gastric Banding, Gastric Bypass, and Gastric Mini-Bypass). We included all subjects that, between January 2015 and December 2020, had undergone a presurgical psychological and psychiatric evaluation at the Psychiatry Unit, “Gaspare Rodolico” Hospital of Catania, Italy.

Before the patients underwent psychiatric assessment, they all received a thorough medical evaluation as part of the pre-surgical assessment to identify potential candidates for bariatric surgery. Patients who were considered suitable for bariatric surgery by the surgeon were referred for consultation with the psychiatrist. Patients who were deemed unsuitable for bariatric surgery due to major psychiatric conditions, as per the criteria outlined in the Diagnostic and Statistical Manual of Mental Disorders, Fifth Edition (DSM-5), were excluded. They were advised to undergo pharmacotherapy or psychotherapy before reapplying. They did not undergo the full psychological tests. For the patients considered eligible for bariatric surgery, we collected demographic information including sex, age, education, height, weight, and medical comorbidities (such as hypertension, type 2 diabetes, thyroid disorders, and obstructive sleep apnea syndrome). Obesity severity was assessed using BMI (kg/m^2^). Finally, the psychologist administered the Eating Disorder Inventory 2 (EDI-2), Beck Depression Inventory-2 (BDI-2), and Minnesota Multiphasic Personality Inventory-2 (MMPI-2) scales. The study conformed to the Committee on Publication Ethics (COPE) guidelines^1^ (22). The manuscript fits with the Uniform Requirements for Manuscripts Submitted to Biomedical Journals and the “Strengthening the Reporting of Observational Studies in Epidemiology” (STROBE) Statement: guidelines for reporting observational studies (23). All the data were collected anonymously and voluntarily. All participants gave their informed consent for data collection and publication. The study was conducted in accordance with the Declaration of Helsinki and was approved by University of Catania Psychiatry Unit review board (n. 2/2020).

### 2.2 Psychological assessment questionnaires

#### 2.2.1 Eating Disorder Inventory 2

The EDI-2 is a self-report questionnaire to evaluate eating disorder symptoms (24). The questionnaire consists of 91 items with a 6-point Likert scale ranging from 1 = “never” to 6 = “always.” The EDI-2 subscales are: Drive for thinness (DT); Bulimic tendencies (B); Body Dissatisfaction (BD); Ineffectiveness (I); Perfectionism (P); Interpersonal Distrust (ID); Interceptive Awareness (IA); Maturity Fears (MF); Asceticism (A); Impulse Regulation (IR) and Social Insecurity (SI) (24). The instrument was validated in an Italian sample, demonstrating good reliability and internal consistency (25).

#### 2.2.2 Beck Depression Inventory-2

The self-report BDI-2 questionnaire evaluates the presence and intensity of depressive symptoms (26). It is the update and improvement of the original BDI. It is composed of 21 items that describe the most frequent depressive symptoms. Each item is graded according to the intensity of each symptom experienced during the last week. Likert options range from 0 (no complaint) to 3 (severe complaint). In addition, the different levels of depression are classified based on the following score cut-offs: absence of depression (0–13), mild depression (14–19), moderate depression (20–29), and severe depression (30–63) (26). The Italian version showed good psychometric properties (27).

#### 2.2.3 Minnesota Multiphasic Personality Inventory-2

The MMPI-2 evaluates several personality characteristics and emotional disorders (28). Consisting of 567 items, it includes three validity scales (Infrequency [F], Lie [L], Defensiveness [K]) to detect whether patients are exaggerating or downplaying psychological symptoms (28); 10 clinical scales (Hypochondriasis [Hs], Depression [D], Hysteria [Hy], Psychopathic deviate [Pd], Masculinity/Femininity [Mf], Paranoia [Pa], Psychasthenia [Pt], Schizophrenia [Sc], Hypomania [Ma], Social introversion [Si]), designed to assess significant categories of abnormal human behavior (16), and 15 content scales (Anxiety [ANX], Fears [FRS], Obsessiveness [OBS], Depression [DEP], Health Concerns [HEA], Bizarre Mentation [BIZ], Anger [ANG], Cynicism [CYN], Antisocial Practices [ASP], Type A Behavior [TPA], Low Self Esteem [LSE], Social Discomfort [SOD], Family Problems [FAM], Work Interference [WRK], Negative Treatment Indicators [TRT]). We did not use the supplementary scales of the MMPI-2. In both the original and Italian validation studies, MMPI-2 showed good reliability and validity (28, 29).

### 2.3 Statistical analysis

We used descriptive statistics to summarize the demographic variables. Continuous data were expressed as mean ± standard deviation, while non-continuous data were expressed as frequency and percentages. The normal distribution of the quantitative variables was evaluated using the Shapiro Wilk test underlined the non-normality of the distribution of some variables. The primary aim of this study was to identify subgroups of patients seeking bariatric surgery using a cluster analysis. The independent variables were the scores on the Eating Disorder Inventory-2 (EDI-2) subscales, while the dependent variables were the scores on the Beck Depression Inventory-2 (BDI-2) and the Minnesota Multiphasic Personality Inventory-2 (MMPI-2) subscales, along with demographic information such as age, gender, weight, and BMI. An exploratory K-means cluster analysis was conducted considering all the EDI-2 subscales to investigate how many main clusters the subjects’ data aggregated. After identifying two main groups, a series of non-parametric Mann–Whitney tests was conducted to identify any differences between the two groups concerning the following variables: BMI, BDI-2, and MMPI-2 subscales scores. A Chi-Square test was used to compare ordinal variables. Yates’ correction for continuity was applied to the Chi-Square test when analyzing 2×2 contingency tables. The Bonferroni correction for multiples comparison was applied. Statistical analyses were carried out using the Statistical Package for the Social Sciences—SPSS 23.0 software (SPSS Inc., Chicago, IL, United States). A *p*-value <0.05 was considered statistically significant.

## 3 Results

This study enrolled 159 adult patients. From the original sample, we could analyze complete data for the primary outcome of 115 subjects. The majority of the sample is composed by females (65.2%), with an average age of 37 years (±7.93), an average body weight of 118.42 kg (±19.42) and a BMI of 42.25 (± 5.6). Other sociodemographic characteristics of the sample are reported in Table 1.

**Table 1:** Sociodemographic features of study subjects.

Variable		*n* = 115
Age (years)		37.0 ± 7.93
Gender	Males Females	40 (34.8) 75 (65.2)
Actual weight (kg)		118.24 (19.42)
Actual body mass index		42.25 (5.6)
Surgical procedure	Lap-band surgery Gastric bypass Sleeve gastrectomy	20 (17.74) 43 (37.1) 52 (45.16)
Educational level	Primary school	4 (3.5)
	Secondary school	72 (62.6)
	High school	35 (30.4)
	University degree	4 (3.5)
Employment	Employed	40 (34.3)
	Unemployed	75 (65.7)

Data are expressed as mean ± standard deviation or as frequencies (percentages).

The K-Means exploratory cluster analysis, based on the scores of the EDI-2 scale, divided the sample into two subgroups. Cluster validation was acceptable, assessed with cohesion and separation as proxy measures. The K-Means exploratory cluster analysis, based on the scores of the EDI-2 scale, identified two psychopathological profiles, which significantly differed in every EDI-2 sub-scale, as reported in Table 2.

**Table 2:** EDI-2 subscales scores in the clinical subgroups.

EDI subscales	More severe (*n* = 39)	Less severe (*n* = 76)
Drive for thinness (DT)	15.10 (3.58)	8.45 (4.94)
Bulimia (B)	5.85 (3.83)	0.99 (2.09)
Body dissatisfaction (BD)	23.18 (4.06)	16.13 (6.63)
Ineffectiveness (I)	8.54 (5.82)	2.86 (3.04)
Perfectionism (P)	5.74 (3.35)	2.63 (2.65)
Interpersonal distrust (ID)	7.03 (4.3)	3.04 (3.07)
Interoceptive awareness (IA)	8.77 (5.92)	2.22 (2.13)
Maturity fears (FM)	8.59 (4.83)	5.74 (4.16)
Asceticism (ASC)	7.31 (3.47)	4.14 (2.65)
Impulse regulation (IR)	5.9 (5.38)	1.61 (2.51)
Social insecurity (SI)	6.23 (4.35)	2.79 (2.41)

Data are expressed as mean ± standard deviation.

The first subgroup is characterized by more severe eating disorder symptoms (*n* = 39), the second one by less severe eating disorder symptoms (*n* = 76), measured by the EDI-2. An ANOVA was conducted to compare BDI scores among different BMI categories: Severe Obesity (BMI ≥ 40), Moderate Obesity (35 ≤ BMI < 40), and Mild Obesity (30 ≤ BMI < 35). The mean BDI scores were 9.33 for Severe Obesity, 10.11 for Moderate Obesity, and 13.67 for Mild Obesity. The analysis showed no statistically significant differences in BDI scores among the categories [*F*(2, N) = 1.757, *p* = 0.178]. Among the MMPI scales, clinical scales revealed elevated scores in the more severe group for Depression (59.9 vs. 53.43), Psychasthenia (56.62 vs. 47.96), and Social Introversion (57.26 vs. 49.08). The validity scale Infrequency was higher in the more severe group (59.59 vs. 49.8), while Defensiveness was lower (42.85 vs. 50.43). Content scales demonstrated consistently higher scores in the more severe group across multiple domains: Anxiety (57.67 vs. 47.74), Fears (55.54 vs. 46.88), Obsessiveness (53.41 vs. 44.46), Depression (59.18 vs. 48.5), Anger (54.21 vs. 44.78), Cynicism (58 vs. 50.09), Type A Behavior (50.23 vs. 43.05), Low Self Esteem (57.03 vs. 45.27), Family Problems (52.23 vs. 45.04), Work Interference (56.64 vs. 45.59), and Negative Treatment Indicators (58.77 vs. 47.36). The only scale scoring significantly lower was the K validity scale (Table 3).

**Table 3:** Comparison of demographic variables and MMPI-2 subscales scores between more severe and less severe EDI-2 clusters.

Variables	More severe (*n* = 39)	Less severe (*n* = 76)
Age	36 (8.59)	37.5 (7.59)
Body mass index	44.44 (6.82)	42.68 (4.8)
Beck depression inventory*	14.7 (6.94)	7.71 (6.64)
Validity scales		
Lie—L Infrequency—F* Defensiveness—K*	54.64 (7.25) 59.59 (12.11) 42.85 (7)	57.76 (11.18) 49.8 (10.87) 50.43 (11.47)
Clinical scales		
Hypochondriasis—Hs	60.1 (8.8)	60.04 (13.19)
Depression—D*	59.9 (8.96)	53.43 (11.31)
Hysteria—Hy	52.13 (9.25)	53.43 (12.54)
Psychopathic deviate—Pd	55.77 (7.97)	53.19 (9.96)
Paranoia—Pa	55.67 (9.47)	50.51 (9.61)
Masculinity/Femininity—MF	56.23 (12.91)	49.54 (10.59)
Psychastehnia—Pt*	56.62 (9.9)	47.96 (8.18)
Schizophrenia—Sc	55.87 (11.12)	50.84 (9.51)
Hypomania—Ma	55.38 (11.2)	49.34 (10.18)
Social introversion—Si*	57.26 (8.53)	49.08 (9.53)
Content scales		
Anxiety—ANX*	57.67 (9.26)	47.74 (10.45)
Fears—FRS*	55.54 (10.77)	46.88 (10.23)
Obsessiveness—OBS*	53.41 (10.9)	44.46 (9.19)
Depression—DEP*	59.18 (10.9)	48.5 (10.56)
Health concerns—HEA	61.36 (11.3)	56.31 (12.64)
Bizarre mentation—BIZ	56.31 (12.91)	49.12 (9.99)
Anger—ANG*	54.21 (11.23)	44.78 (10.93)
Cynicism—CYN*	58 (9.58)	50.09 (11.15)
Antisocial practices—ASP	55.08 (9.92)	49.36 (11.11)
Type A behavior—TPA*	50.23 (11.27)	43.05 (11.37)
Low self-esteem—LSE*	57.03 (10.74)	45.27 (8.09)
Social discomfort—SOD	51.28 (9.51)	46.73 (9.18)
Family problems—FAM*	52.23 (9.82)	45.04 (10.11)
Work interference—WRK*	56.64 (10.66)	45.59 (10.27)
Negative treatment indicators—TRT*	58.77 (11.21)	47.36 (10.72)

Data are expressed as mean ± standard deviation. *p < 0.05 after Bonferroni correction.

A chi-square test was conducted to compare the prevalence of various health conditions between the two clusters. The conditions analyzed included hypertension, diabetes type II, thyroid disorders, and OSAS. The results indicated no statistically significant differences between the clusters for any of the conditions. For details refer to Table 4.

**Table 4:** Comparison of medical conditions between the more and less severe clusters.

Condition	Presence	More severe (*n* = 39)	Less severe (*n* = 76)	Yates’ chi-square	*p*-value
Hypertension	No	23	47	0.0093	0.923
	Yes	16	29		
Diabetes type II	No	26	55	0.175	0.676
	Yes	13	21		
Thyroid disorders	No	27	54	0.0	1.0
	Yes	12	22		
OSAS	No	33	61	0.100	0.751
	Yes	6	15		

OSAS, Obstructive Sleep Apnea Syndrome.

## 4 Discussion

This study aimed to identify clinical subgroups of bariatric surgery candidates with the k-means clustering method using the EDI-2 scale, BDI-2 scale, and MMPI-2 scale. Results indicate that our sample of bariatric surgery candidates can be classified into two groups based on the severity of their eating disorders. The most severe cluster had higher scores in the BDI-2 and MMPI-2 content scales.

In this regard, elevated scores on the depression, psychasthenia, and social introversion scales may reflect underlying emotional distress and social difficulties that may exacerbate eating disorder symptoms. Similarly, higher scores on the anxiety, fears, obsessiveness, anger, cynicism, type A behavior, and low self-esteem scales may indicate maladaptive coping mechanisms or personality traits that may contribute to the development or maintenance of eating disorders.

The cluster analysis indicates that the severity of the eating disorder is associated with higher psychopathology, particularly in terms of depression and specific personality dimensions. Individuals in the more severe group exhibited elevated scores on several MMPI-2 scales, reflecting a complex pattern of psychological distress. Notably, they showed higher levels of social introversion, suggesting difficulties in interpersonal relationships and a tendency to withdraw from social interactions. The elevated psychasthenia scores point to increased anxiety, obsessive thoughts, and feelings of fear and apprehension. Additionally, the more severe group demonstrated higher levels of cynicism and anger, which may manifest as interpersonal mistrust and difficulty managing emotions. Low self-esteem emerged as another significant factor, potentially contributing to and exacerbating the eating disorder symptoms. The higher scores on work interference and family problems scales suggest that the psychological distress extends beyond individual symptoms, impacting various life domains.

Our results align with prior reports showing a pivotal role of depression in obese individuals seeking treatments (30, 31). For instance, a previous study indicated a correlation between the severity of depression, emotional/external eating behaviors, and restrictive eating behaviors (32). In addition, depressive symptoms in pre-bariatric surgery patients are associated with poor body image, overvaluation of shape and weight (OSW), and eating disorder psychopathology (33, 34). To which extent depression affects long-term post-surgical outcomes remains to be investigated. According to a recent large cohort study, one-third of bariatric surgery candidates had preoperative depression and showed similar outcomes vs. non-depressed patients (weight loss, diabetes, and safety/utilization outcomes (35). Of note, published results regarding depression as a potential mediator of poor outcomes after bariatric surgery are heterogeneous (36). Thus, an optimal presurgical evaluation of depression and related factors is important in bariatric surgery candidates. Our results support this hypothesis and provide additional evidence for homogeneous subgroups of patients according to the severity of their eating disorder, mood, and related cognitive/ behavioral dimensions. As previously noted, the use of both MMPI-2 and BDI-2 provides a better assessment of depressive symptomatology and associated psychopathology (21). For instance, we found that the patients in the more severe cluster reported higher scores in Pt, ANX, FRS, and OBS content scales of MMPI-2. The Pt scale identifies “psychasthenia” as a characteristic depressive profile associated with anxiety, inhibition, and phobic or obsessive components. Thus, we show that in the more severe cluster, the most common depressive symptoms include pessimism, hypersensitivity to rejection, low sense of autonomy, rumination, and lack of self-confidence. Rumination and retrieval of negative memories alter cortical activation (37) and are associated with weight gain (38). The ANX, FRS, and OBS content scales are related to somatic anxiety, specific phobias, and obsessive thinking. In keeping with previous studies, our data indicate that in the more severe cluster, the obsessive spectrum, social anxiety, and maladaptive perfectionism add to the depressive symptomatology (39). Stress and anxiety disrupt cortical plasticity in humans (40, 41), and these changes in neural plasticity induced may play a significant role in the development of depression. The relationship between presurgical anxiety and depression comorbidities and their possible role as mediators of poor outcomes after bariatric surgery must be addressed in future longitudinal studies (31, 32).

Individuals in the more severe cluster displayed higher scores in the ANG, CYN, and TPA content scales of MMPI-2. These dimensions are related to externalizing symptoms, such as anger and hostility. They are often associated with mistrust, suspiciousness, interpersonal sensitivity, and a tendency toward persecutory ideation in response to stressful situations. Our results are consistent with previous studies which used different instruments and demonstrated higher anger, fear, and hostility in bariatric surgery candidates (42, 43). The low self-esteem in the more severe cluster (higher LSE content scale of MMPI-II) we reported is also consistent with the literature indicating that self-criticism, self-disgust, and shame are often associated with eating disorders, eating stress, and maladaptive coping style (44). Bariatric candidates with more severe eating disorders and depressive symptoms showed higher scores in the FAM, WRK, and Si content scales of MMPI-2. These elements indicate conflicting family relationships, problems in the work environment, and marked isolation, and relate to stigma and loneliness often reported in patients with eating disorders (45, 46). Overall, the patient clusters identified in this study could be used as a clinical phenotyping integrating psychiatric comorbidity and severity of eating disorders in obese patients that may be used more broadly, not only for the presurgical evaluation. Hence, the MMPI-2 content scales seemed helpful in comprehensively assessing symptoms associated with mental illness or a personality disorder previously addressed using different scales in obese patients seeking surgery.

Several study limitations deserve mention. First, excluding patients with major psychiatric disorders ensured a homogeneous study population but limits the applicability of our findings to the broader population of bariatric surgery candidates with comorbid psychiatric conditions. Second, although the EDI-2 and the MMPI-2 scales are still widely used, there are now shorter versions of these scales that might be administered more easily (47, 48). Third, the cross-sectional nature of this study prevents discussing a direct causal link between the variables, which should be investigated in longitudinal studies. Future research could benefit from exploring the interplay between eating disorder severity, depression, and personality dimensions in the two identified clusters to investigate the impact of these psychological factors on surgical outcomes, adherence to subsequent treatments, and long-term weight management. Furthermore, in this study, we did not collect biological measures (e.g., blood tests) because these data were not available in our outpatient clinic. The complex nature of the psychopathology of obesity and eating disorders implies that future studies must confirm and expand our findings using different instruments and biological measures.

## 5 Conclusion

The cluster analysis allowed identifying two different subgroups of bariatric surgery candidates. Using the BDI-2 and MMPI-2 scales provided incremental validity in detecting depressive symptoms and associated behavioral comorbidity. Thus, this research provides valuable insight into the psychopathological aspects of this sample of patients. Investigating the interplay between the severity of the eating disorder, depression, and personality dimensions in the two clusters may be advantageous in improving quality of life, patient management, and treatments outcome.

## Data availability statement

The raw data supporting the conclusions of this article will be made available by the authors upon reasonable request.

## Ethics statement

The study was conducted in accordance with the Declaration of Helsinki and was approved retrospectively by the University of Catania Psychiatry Unit review board (n. 2/2020). The studies were conducted in accordance with the local legislation and institutional requirements. The participants provided their written informed consent to participate in this study.

## Author contributions

AR: Conceptualization, Validation, Visualization, Writing – original draft, Writing – review & editing, Data curation, Formal analysis, Methodology, Project administration. VR: Methodology, Project administration, Visualization, Writing – original draft, Writing – review & editing, Investigation, Conceptualization, Data curation. CR: Conceptualization, Data curation, Visualization, Writing – original draft, Formal analysis. CC: Conceptualization, Writing – original draft, Methodology. VM: Writing – original draft, Investigation. GS: Investigation, Writing – original draft, Validation. SS: Investigation, Validation, Writing – original draft. MS: Project administration, Supervision, Visualization, Writing – review & editing. RW: Writing – original draft, Data curation, Validation. RS: Validation, Writing – original draft, Visualization. CP: Validation, Writing – original draft, Data curation. MK: Data curation, Validation, Writing – original draft. AE: Project administration, Supervision, Visualization, Writing – original draft. ST: Supervision, Visualization, Writing – original draft. FT: Supervision, Visualization, Writing – review & editing. FB: Conceptualization, Supervision, Validation, Visualization, Writing – original draft, Writing – review & editing.

## Footnotes

^1^ http://publicationethics.org
